# Development of a Lateral Flow Assay for the Detection of the Hepatitis C Virus Core Antigen

**DOI:** 10.3390/ph17081022

**Published:** 2024-08-04

**Authors:** Erick Joan Vidal-Alcántara, Sonia Hernández Antón, Paloma Rueda, María Belén Yélamos, Julián Gómez, Salvador Resino, Alba Fresco-Taboada, Isidoro Martínez

**Affiliations:** 1 Unidad de Infección Viral e Inmunidad, Centro Nacional de Microbiología, Instituto de Salud Carlos III, Majadahonda, 28220 Madrid, Spain; ejvidal@isciii.es; 2 Gold Standard Diagnostics Madrid S.A (GSD Madrid), Calle de los Hermanos García Noblejas, 39, 28037 Madrid, Spain; sonia.hernandezanton@eu.goldstandarddiagnostics.com (S.H.A.); paloma.rueda@eu.goldstandarddiagnostics.com (P.R.); alba.fresco@eu.goldstandarddiagnostics.com (A.F.-T.); 3 Departamento de Bioquímica y Biología Molecular, Facultad de Ciencias Químicas, Universidad Complutense, 28040 Madrid, Spain; mbyelamos@quim.ucm.es (M.B.Y.); jgomezgu@ucm.es (J.G.); 4 Centro de Investigación Biomédica en Red en Enfermedades Infecciosas (CIBERINFEC), Instituto de Salud Carlos III, 28029 Madrid, Spain

**Keywords:** hepatitis C, core antigen, monoclonal antibody, rapid diagnostic test

## Abstract

Background: Hepatitis C virus (HCV) infection remains a global health challenge, with millions of people affected annually. Current diagnostic methods, reliant on antibody screening and viral RNA detection, are complex, costly, and often inaccessible, particularly in resource-limited settings. Aim: Development of a lateral flow immunochromatography-based assay for detecting the highly conserved hepatitis C core antigen (HCVcAg). Methods: The assay relies on the interaction of four highly specific and cross-reactive monoclonal antibodies with recombinant HCVcAg from five different genotypes in a double antibody sandwich format. Latex and colloidal gold were evaluated as detector nanoparticles. Results: Extensive evaluation of 32 antibody combinations led to identifying the most sensitive antibody pairs. The chosen assay, named LN17, demonstrated a target sensitivity of 10 ng/strip, with potential clinical implications for detecting HCV. Furthermore, the study examined matrix effects in serum samples, providing valuable insights for future clinical application. Conclusions: The developed assay holds promise as a rapid, cost-effective, and user-friendly tool to enhance accessibility to hepatitis C screening, especially in high-risk populations and resource-limited environments.

## 1. Introduction

Hepatitis C virus (HCV) infection represents a significant global health challenge. According to the World Health Organization (WHO), an estimated 50 million people have chronic hepatitis C worldwide, with 1 million new infections occurring yearly [1]. Hepatitis C can lead to severe complications such as cirrhosis, end-stage liver disease, or hepatocellular carcinoma [2]. In 2022, approximately 242,000 deaths were attributed to complications from HCV infection [1].

HCV is primarily transmitted through contact with infected blood. This can occur through injection drug use, blood transfusions, unsafe medical procedures, and sexual practices that cause bleeding [2]. This places specific populations, including injecting drug users (IDUs) and individuals engaging in activities with bleeding risk, particularly men who have sex with men (MSM), at higher risk of infection [3].

While no HCV vaccine is available, direct-acting antivirals (DAAs) offer highly effective treatment, curing over 95% of HCV-infected patients [4]. Based on this, the WHO aims to eliminate hepatitis C as a public health concern by 2030, targeting the diagnosis of 90% of HCV-infected individuals and treating 80% of them [5]. However, due to the often asymptomatic nature of hepatitis C for several years after initial infection, around 64% of infected individuals are unaware of their status and, consequently, do not receive HCV treatment [6]. Achieving the WHO target requires significantly increasing the number of people diagnosed by large-scale screening efforts, especially in developing countries and among high-risk populations [7]. However, many of these individuals have limited access to health services, making the screening process challenging.

The current standard diagnosis for hepatitis C involves an initial HCV antibody screening test, followed by a second viral RNA detection test for confirmation in cases of a positive result [1,8]. This methodology is complex, expensive, and demands specialized laboratory equipment, trained personnel, and time. Consequently, its application at the population level and among hard-to-reach risk groups is limited [9]. Therefore, there is a pressing need to develop a rapid, affordable, and user-friendly diagnostic test that can be administered at patient care points or for self-diagnosis. Several tests meet these criteria but only detect anti-HCV antibodies. However, anti-HCV tests can detect both current and past HCV infections but cannot distinguish between them. Individuals with positive anti-HCV test results must also be tested with another assay (currently a nucleic acid test, NAT) to determine the infection status.

HCV core antigen (HCVcAg) is a highly conserved protein across various HCV genotypes [10,11] and, like viral RNA, is detectable in the blood of actively infected patients [12,13]. Additionally, HCVcAg levels strongly correlate with viral RNA levels [14], making HCVcAg detection suitable for HCV screening and diagnosis. Tests based on HCVcAg detection are potentially cheaper, faster, and easier to use than those detecting viral RNA, offering improved access to hepatitis C screening, particularly for at-risk populations [15]. While tests for HCV RNA or HCVcAg detection already exist or are undergoing approval [16], none fully meet all the desired requirements for point-of-care or self-diagnostic applications, such as rapidity, low cost, and ease of use.

Lateral flow assays (LFA) are easy to use, cost-effective, provide rapid results, and are stable in various climates, making them one of the most widely used techniques for point-of-care testing [17,18]. In this study, we developed a lateral flow diagnostic assay using four monoclonal antibodies previously obtained in our laboratory. These antibodies react with HCVcAg from different genotypes [19]. The assay was evaluated by testing recombinant HCVcAg and served as a proof of principle, demonstrating the utility of these antibodies in LFA.

## 2. Results

To produce the detector reagents, the four described antibodies specific to the core antigen of the hepatitis C virus (HCVcAg) were coupled to nanoparticles, latex, and colloidal gold. Furthermore, they were also dispensed onto the nitrocellulose membrane to act as capture reagents. A total of 32 combinations were obtained when cross-testing all the detectors with all the capture reagents, 16 belonging to the latex group and 16 to the colloidal gold group (Supplementary Table S1).

First, the running buffer was tested to discard those pairs that yielded improper results. Four out of the thirty-two pairs led to false positive results (Supplementary Table S1). Hence, 28 combinations were selected to analyze the recombinant proteins. To make a first screening, HCVcAg from H77 (Gt1a) was analyzed at different concentrations, selecting the combinations that gave a positive or weak positive result of at least 100 ng/ml. Based on this, six latex and three colloidal gold combinations, which involved all the four antibodies, were selected (Table 1).

Afterward, all the described HCVcAgs were 2-fold serial diluted to determine the selected pairs’ limit of detection (LoD). As observed in Table 1, although it is protein-dependent, the most sensitive combinations used latex nanoparticles (LN) as tracers: LN3, LN4, and LN5.

Even if MAb 8C did not recognize Gt2a and slightly recognized Gt3a in Western blot [19], both HCVcAgs were detected by the pairs that contained 8C MAb. On the other hand, 2C is the antibody with the strongest binding properties; none of the combinations in which it was involved were included in the most sensitive selected combinations, confirming the hypothesis that it is not suitable for a double antibody sandwich assay [19].

Since antibodies 1C, 2C, and 4C competed with each other but not with 8C, and based on the results obtained, a new LN test based on LN3 and LN4 was conceived, named LN17, involving 8C and 4C as detectors and maintaining 4C as capturer. The sensitivity was increased with most of the HCVcAgs, and it was selected as the most promising test.

**Figure 1:**
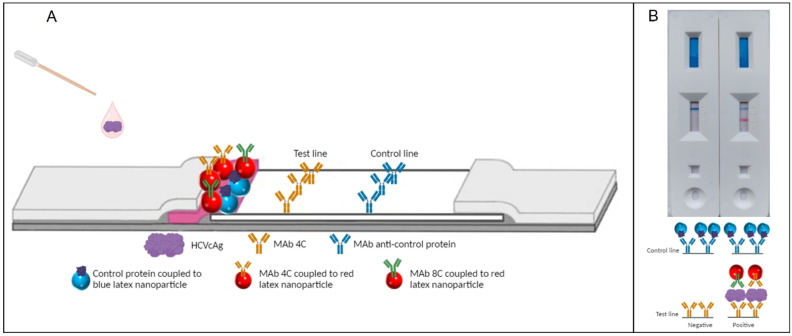
Schematic representation of the LN17 lateral flow assay for detecting HCVcAg. (**A**) Scheme of the components of the developed lateral flow assay. (**B**) Picture of a negative and a positive lateral flow assay; representation of the antibody-antigen complex presence or absence in the test line.

In this test, LN17, the membrane contains a test line in which 4C MAb was immobilized and a control line in which an antibody to the control protein was immobilized. Furthermore, red latex nanoparticles coated with both 4C and 8C MAb were dispensed in the conjugate pad, along with blue latex particles coated with the control protein (Figure 1A).

When the sample containing HCVcAg is added to the sample pad, the HCVcAg will interact with the antibodies bound to the surface of the red latex nanoparticles (Figure 1A). After adding the running buffer, these latex-antibody-antigen complexes will migrate through the membrane and bind to the MAb 4C immobilized in the test line. If no HCVcAg is present in the sample, no color will appear in the test line. In contrast, if the sample is positive, the antigen captured by the double antibody sandwich will result in a reddish color in the test line. In both cases, blue latex nanoparticles covered with the control protein will migrate through the membrane and be recognized by the monoclonal antibody immobilized in the control line (Figure 1B). For this reason, the appearance of a blue line in the control region indicates that the assay developed correctly.

**Figure 2:**
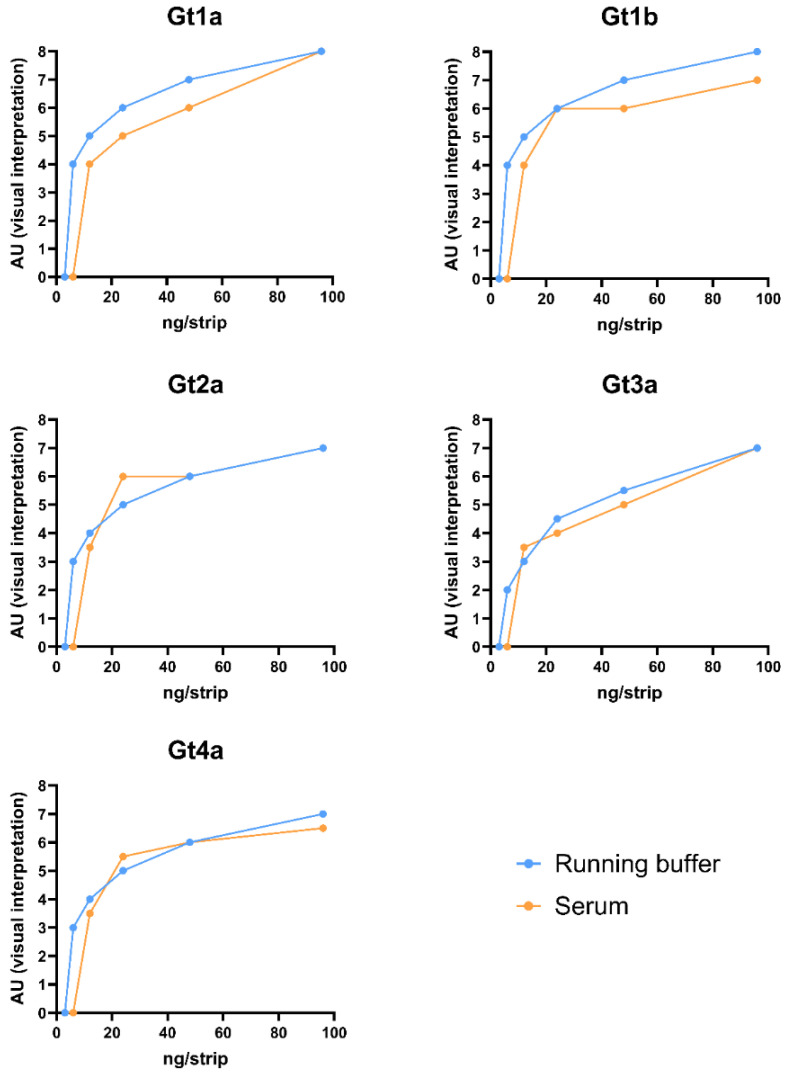
Matrix effect: Comparison of serial dilutions of HCVcAg from various HCV genotypes, either diluted in running buffer or spiked in negative serum. The combination LN17 was used in the experiments. Cut-off: ≥4 AU (arbitrary units).

Test LN17 was selected to study if a matrix effect could be observed using serum samples. The HCVcAgs were serially diluted in a negative serum sample and tested, adding 10 μl of the serum and 110 μl of running buffer. As shown in Figure 2, the sensitivity decreased by a 2-fold dilution when analyzing most genotypes. For HCVcAg Gt1a and Gt1b, the LoD was 6 ng/strip in the running buffer (Table 1) and 12 ng/strip in the serum. For Gt2a and Gt4a, it ranged from 12 ng/strip in the running buffer to 24 ng/strip in serum. In contrast, the LoD for HCVcAg Gt3a was maintained at 24 ng/strip in both buffer and serum. This detection limit was calculated based on the T line intensity determined using the signal intensity scale, where a value of 4 AU corresponds to a weak positive result.

## 3. Discussion

This study is a proof of principle demonstrating that the antibodies against the HCV core antigen (HCVcAg) previously obtained in our laboratory [19] are viable for application in a lateral flow assay. Developing a lateral flow assay to detect HCVcAg addresses a critical need in hepatitis C management. HCV infection poses a substantial public health challenge, with millions affected worldwide, and its associated complications can be severe and life-threatening [2]. The existing diagnostic methods, involving antibody screening followed by viral RNA detection, are complex, expensive, and often inaccessible for high-risk populations and in resource-limited settings.

Focusing on HCVcAg detection is promising due to its high conservation across various HCV genotypes and its correlation with viral RNA levels [14]. This presents an opportunity to develop a diagnostic tool that is potentially faster, more affordable, and user-friendly compared to RNA-based tests. This study takes advantage of the stability and accessibility of lateral flow assays (LFAs) as a platform, which are well-established for point-of-care testing [20,21,22]. Although there are HCVcAg-based tests available, they do not fulfill all the requirements for point-of-care use [16].

The selection and characterization of four monoclonal antibodies (MAbs) against HCVcAg [19] and the choice of latex and colloidal gold nanoparticles as detector reagents represent a comprehensive approach to assay development. The subsequent combination of these elements in a double antibody sandwich (DAS) format on a lateral flow strip leverages the advantages of LFAs for POC applications. The extensive testing of 32 combinations, followed by the selection of the most effective ones based on their performance in detecting HCVcAg is a critical aspect of this study. The sensitivity achieved with specific combinations using latex nanoparticles as tracers is promising, especially in the case of chronically infected individuals who typically have high serum HCV-RNA levels exceeding 10^6^ copies/ml [23]. Moreover, this sensitivity can be improved further through assay optimization, signal amplification techniques, and the use of fluorescent nanoparticles [18,24,25].

The evaluation of the assay’s performance with HCVcAg diluted in a negative serum sample and the observation of a slight decrease in sensitivity highlight the importance of understanding potential matrix effects. This consideration is crucial for clinical application, especially given that the intended use involves blood or serum samples. These samples might contain antibodies against HCVcAg, which could interfere with the assay’s performance by hindering their interaction with the antibodies present on the test strip. While adding immune-complex dissociating detergents to the samples may mitigate this problem [26], it is expected that the test’s performance would be improved for detecting HCVcAg in the preseroconversion period of a recent infection, which represents a big gap in routine diagnosis [27,28,29].

### Limitations and Future Directions

The study’s main limitation is that the tests were not validated in clinical samples. This validation is essential to confirming the assay’s clinical utility. Gathering a large number of clinical samples from HCV-infected patients to determine the sensitivity and specificity and to check the robustness of the assay precisely is challenging. However, we are in contact with different hospitals to achieve this goal. Second, the LN17 test demonstrated increased sensitivity for most HCV genotypes. However, the limit of detection (LoD) was slightly higher for Gt3a compared to other genotypes. This could be relevant when testing intravenous drug users, as this population may have a higher prevalence of Gt3a [30]. 

Developing an LFA assay for HCVcAg detection is gaining increasing attention, as evidenced by the growing number of publications in this field [31,32]. Furthermore, in addition to antibodies, other recognition molecules, particularly aptamers, are being investigated as potential detectors of HCVcAg [33,34]. Finally, there is a need to increase the sensitivity and specificity of the assays, a process which is driven by continuous advancements in techniques for assay optimization and signal amplification [18,24].

## 4. Materials and Methods

Monoclonal antibodies (MAb) specific against HCV core antigen (HCVcAg) described by Vidal-Alcántara et al. (2023) [19], named 1C, 2C, 4C, and 8C, were used as capture or detector reagents for the development of a lateral flow assay in a double antibody sandwich (DAS) format. As detector nanoparticles, latex, and colloidal gold were used. We tested each detector with every capture reagent, yielding a total of 32 different combinations (16 for latex detectors and 16 for colloidal gold detectors) (Supplementary Table S1).

### 4.1. Capture Reagent

Each MAb was diluted to 0.5–1.5 mg/mL in 20 mM Tris-HCl buffer at pH 7.5 to be used as the test line capture reagent. A monoclonal antibody against a control protein (Operon, Cuarte de Huerva, Spain) at 1 mg/mL was used as a control line capture reagent. Both reagents were dispensed in 2 parallel lines on an HF120 nitrocellulose membrane (Merck KGaA, Darmstadt, Germany). After drying for 5 min at 45 °C, the membranes were sealed and stored at room temperature under low-humidity conditions.

### 4.2. Detector Reagent

#### 4.2.1. Latex Nanoparticles

MAbs were conjugated to latex nanoparticles by covalent attachment. Briefly, 300 nm red latex beads were activated with 1-ethyl-3-(3-dimethylaminopropyl) carbodiimide hydrochloride (EDC) and N-hydroxysuccinimide (NHS) (Sigma-Aldrich, San Louis, MO, USA) and then coupled to each MAb at a surface concentration of 1 mg/m^2^. Blue latex beads (Ikerlat Polymers S.L., Lasarte-Oria, Spain) were conjugated with the control protein (BSA-Biotin) following the same protocol.

To prepare the conjugate solution, the Mab-latex and control–latex particles were diluted at a concentration of 0.10–0.15% each in a 25 mM Tris-HCl pH 9.5 buffer. The mixture was dispensed onto the conjugate pad (Operon, Cuarte de Huerva, Spain), dried for 30 min at 45 °C, and stored at room temperature under low-humidity conditions.

#### 4.2.2. Colloidal Gold

MAbs were conjugated to colloidal gold (Nanoflow, Liège, Belgium) by passive adsorption. First, antibody concentration and pH were titrated to determine the optimal conditions for colloidal gold stabilization. Then, colloidal gold was diluted to 1 O.D. in the optimal pH, and the optimal quantity of MAb was added to the suspension. After a 30-minute incubation, the mixture was centrifuged, and the conjugated gold was resuspended in the initial buffer volume. If necessary, a blocking step with 2% BSA was also performed before centrifugation.

### 4.3. Preparation of Lateral Flow Strips

To assemble the 30 cm card, the nitrocellulose membrane (Merck KGaA, Darmstadt, Germany), conjugate pad (Operon, Cuarte de Huerva, Spain), sample pad (Ahlstrom-Munksjo Filtration LLC, Holly Springs, LA, USA), and wicking pad (Ahlstrom-Munksjo Filtration LLC, Holly Springs, LA, USA) were pasted on a plastic backing with adhesive (Shanghai WeiYu Biotechnology Co., Ltd., Shanghai, China) and covered with a cover tape (Ahlstrom-Munksjo Filtration LLC, Holly Springs, LA, USA). The final card was then cut into strips of 4.2 mm width. Each strip was placed inside a plastic cassette (Plásticos Morte, El Burgo de Ebro, Spain) to improve the immunochromatographic flow and to make the final device easier to handle for the user.

### 4.4. Evaluation of Antibody Pairs

Recombinant Hepatitis C virus core proteins (HCVcAgs) expressed in *E. coli* [19] were serially diluted two-fold, starting at 0.8 ng/μL and ending at 25 pg/μL, in a running buffer (250 mM Tris-HCl pH 7.5, 500 mM NaCl, casein, and NaN_3_) to study the limit of detection (LoD) of each test. A 120 μL sample of each dilution was applied to the sample pad, and results were read after 10 min. The proteins belonged to different genotypes (Gt): Gt1a (H77), Gt1b (J4), Gt2a (JFH1), Gt3a (S52), Gt4a (ED43). These HCVcAgs can form multimers, particularly dimers. This characteristic makes them particularly well-suited for assays that rely on the formation of an antibody–antigen–antibody complex. Furthermore, a truncated form of Gt1a (H77, 125 amino acids) was also evaluated.

### 4.5. Matrix Effect Evaluation of Spiked Samples

The final test was designed to be used with serum samples. Once the most promising combination (LN17) was selected, a negative serum (Single donor human serum off the clot; Innovative Research, Peary Court, MI, USA) was spiked with 96 ng/strip of HCVcAg, and serial dilutions up to 3 ng/strip were prepared.

Briefly, 10 μL of spiked negative serum was applied to the sample pad, followed by 110 μL of running buffer (250 mM Tris-HCl pH 7.5, 500 mM NaCl, casein, and NaN_3_). The results were interpreted 10 min after running buffer addition. A signal intensity scale of the test line was used to assign a value from 1 to 10 arbitrary units (AU), giving a semiquantitative value for statistical purposes. The following cut-off values were considered: 1–2 negative, 3 doubtful, 4 weak positive, and 5–10 positive.

## 5. Conclusions

Developing a lateral flow assay for HCVcAg detection is a significant step towards addressing the challenges associated with hepatitis C diagnosis. The rational selection of antibodies, the strategic use of LFAs, and the meticulous evaluation of combinations contribute to the robustness of the assay. Moving forward, clinical validation and potential integration into screening programs are crucial steps toward realizing the goal of eliminating hepatitis C as a public health concern by 2030.

## Figures and Tables

**Table 1:** Limit of detection (LoD) in ng/strip of the different antibody pairs with each recombinant HCVcAg. HCVcAg genotypes were all added to strips in 120 μL aliquots at amounts (ng/strip) listed in the table. LN: latex nanoparticles. CG: colloidal gold.

Combination	Detector Antibody	Capture Antibody	Gt1a-125 ng/strip	Gt1a ng/strip	Gt1b ng/strip	Gt2a ng/strip	Gt3a ng/strip	Gt4a ng/strip
LN1	4C	2C	12	24	>96	48-96	12	12
LN2	8C	2C	6	24	48–96	48-96	12	12
LN3	4C	4C	6	12	24	24	6	24
LN4	8C	4C	6	6	48	96	6	12
LN5	1C	8C	6	12	24	96	6	6
LN6	2C	8C	6	24	48	>96	12	6
CG1	8C	2C	12	12	24	>96	12	24
CG2	8C	4C	12	12	24	96	12	12
CG3	4C	8C	12	24	24	96	6	12
LN17	8C-4C	4C	6	6	6	12	24	12

## Data Availability

The manuscript contains all the relevant information.

## Supplementary Materials

The following supporting information can be downloaded at: https://www.mdpi.com/article/10.3390/ph17081022/s1, Table S1: Combinations obtained after cross-testing the 4 antibodies and the two types of nanoparticles.

## List of Abbreviations

Aa	Amino acids
AU	Arbitrary units
CG	Colloidal gold
CHC	Chronic hepatitis C
DAAs	Direct-acting antivirals
DAS	Double antibody sandwich
EDC	1-ethyl-3-(3-dimethylaminopropyl) carbodiimide hydrochloride
Gt	Genotype
IDUs	Injecting drug users
HBV	Hepatitis B virus
HCl	Hydrochloric acid
HCV	Hepatitis C virus
HCVcAg	Hepatitis C virus core antigen
LN	Latex nanoparticles
LoD	Limit of detection
Mab	Monoclonal antibodies
MSM	Men who have sex with men
NHS	N-hydroxysuccinimide
O.D.	Optical density
PCR	Polymerase chain reaction
RDAT	Rapid diagnostic antigen tests
RNA	Ribonucleic acid
Tris	Tris(hydroxymethyl)aminomethane
WHO	World Health Organization
