# Supplementary material for: Development of a Lateral Flow Assay for the Detection of the Hepatitis C Virus Core Antigen
Source: Pharmaceuticals (Basel). 2024 Aug 4;17(8):1022. doi: 10.3390/ph17081022 (PMC11356972; doi:10.3390/ph17081022)
Supplement: Supplementary file 1 [file pharmaceuticals-17-01022-s001.zip › pharmaceuticals-3134186-supplementary.pdf]

Supplementary Material

Table S1. Combinations obtained after cross-testing the 4 antibodies and the two types of nanoparticles.

Combination	Detector antibody	Capture antibody	Running buffer	200 ng/strip Gt1a	100 ng/strip Gt1a
LN1	4C	2C	negative	positive	positive
LN2	8C	2C	negative	positive	positive
LN3	4C	4C	negative	positive	positive
LN4	8C	4C	negative	positive	positive
LN5	1C	8C	negative	positive	positive
LN6	2C	8C	negative	positive	positive
LN7	1C	1C	negative	doubtful	NA
LN8	2C	1C	negative	negative	negative
LN9	4C	1C	negative	positive	wpositive
LN10	8C	1C	negative	positive	wpositive
LN11	1C	2C	negative	positive	doubtful
LN12	2C	2C	negative	positive	doubtful
LN13	1C	4C	negative	negative	negative
LN14	2C	4C	negative	negative	negative
LN15	4C	8C	FP	NA	NA
LN16	8C	8C	FP	NA	NA
CG1	8C	2C	negative	positive	positive
CG2	8C	4C	negative	positive	positive
CG3	4C	8C	negative	positive	positive
CG4	1C	1C	negative	negative	NA
CG5	1C	2C	negative	negative	NA
CG6	1C	4C	negative	negative	NA
CG7	1C	8C	negative	negative	NA
CG8	2C	1C	negative	negative	NA
CG9	2C	2C	negative	negative	NA
CG10	2C	4C	negative	negative	NA
CG11	2C	8C	negative	negative	NA
CG12	4C	1C	negative	negative	NA
CG13	4C	2C	negative	negative	NA
CG14	4C	4C	negative	negative	NA
CG15	8C	1C	FP	NA	NA
CG16	8C	8C	FP	NA	NA
